# Cumulative exposure and patterns of the cholesterol–HDL-C–glucose index and risk of new-onset cardiometabolic multimorbidity: 9-year longitudinal evidence from CHARLS

**DOI:** 10.3389/fnut.2026.1835874

**Published:** 2026-05-25

**Authors:** Shuxiang Li, Lichan Tao, Danni Wu, Xiaoming Ma, Xiaomin Luo

**Affiliations:** 1 Department of Articular Orthopaedics, The Third Affiliated Hospital of Soochow University, Changzhou, Jiangsu, China; 2 Department of Cardiovascular Medicine, The Third Affiliated Hospital of Soochow University, Changzhou, Jiangsu, China; 3 Department of Endocrinology & Metabolism, The Third Affiliated Hospital of Soochow University, Changzhou, Jiangsu, China; 4 Department of Neurology, Suzhou Hospital, Affiliated Hospital of Medical School, Nanjing University, Suzhou, Jiangsu, China; 5 Department of Neurology, Nanjing University Medical School Affiliated Nanjing Drum Tower Hospital, Nanjing, China; 6 Suzhou Key Laboratory of Integrated Stroke Prevention, Treatment and Rehabilitation, Suzhou, China

**Keywords:** cardiometabolic multimorbidity, CHARLS, cholesterol, glucose, HDL-C

## Abstract

**Background:**

The cholesterol–HDL-C–glucose (CHG) index has been proposed as an integrated marker of glucose–lipid metabolic dysfunction. However, the associations of long-term CHG exposure and its temporal patterns with new-onset cardiometabolic multimorbidity (CMM) remain unclear, particularly among middle-aged and older adults.

**Methods:**

We used data from the China Health and Retirement Longitudinal Study (CHARLS) and included participants aged 45 years or older without baseline CMM. Cumulative CHG (cuCHG) was calculated as a cumulative exposure metric based on CHG measured in 2012 and 2015. K-means clustering was applied to identify broad CHG patterns based on the two repeated measurements. Cox proportional hazards models were used to estimate hazard ratios (HRs) and 95% confidence intervals (CIs), and restricted cubic spline (RCS) analyses were performed to examine the dose–response relationship.

**Results:**

Among 6,515 participants, 593 new-onset CMM events (9.1%) occurred over a median follow-up of 9 years. In the fully adjusted model, participants in the highest quartile of cuCHG had a 78% higher risk of new-onset CMM than those in the lowest quartile (HR 1.78, 95% CI 1.39–2.29), and each 1-unit increase in cuCHG was associated with a 23% higher risk (HR 1.23, 95% CI 1.15–1.32). Three distinct CHG patterns based on two repeated measurements were identified, and only the persistently high CHG pattern group was significantly associated with an increased risk of new-onset CMM (HR 2.05, 95% CI 1.61–2.61). RCS and piecewise Cox analyses suggested a non-linear association, with a potential threshold at a cuCHG value of 14.92. These findings remained robust across subgroup and sensitivity analyses. Compared with baseline CHG, the triglyceride–glucose index, and the atherogenic index of plasma, cuCHG and CHG pattern groups showed better discriminatory performance.

**Conclusions:**

Higher cumulative CHG exposure and a persistently high CHG pattern group were independently associated with an increased risk of new-onset CMM in middle-aged and older Chinese adults. Compared with a single baseline CHG measurement, cumulative CHG exposure and CHG patterns based on repeated measurements may provide additional information for CMM risk stratification.

## Introduction

Cardiometabolic multimorbidity (CMM) is defined as the concurrent presence of at least two cardiometabolic conditions, including, but not limited to, diabetes, heart disease and stroke. It is evident that, as a consequence of population aging, CMM has become a matter of significant global public health concern. CMM is prevalent among Chinese adults, with a marked increase in prevalence with age, and an upward trend has been observed over time. This has important prognostic implications, including an increased risk of all-cause mortality (1–4). In a longitudinal study of over one million Chinese adults, the prevalence of CMM rose from 2.41% to 5.94% over approximately 5 years of follow-up, and the presence of two or three cardiometabolic diseases was associated with a higher risk of mortality compared with a single condition (5). Similar patterns have been reported in other populations. For instance, the prevalence of CMM among US adults increased steadily from 1999 to 2018, reaching 14.4% in 2017–2018, disproportionately affecting older adults (6). Taken together, these findings suggest that CMM substantially contributes to adverse health outcomes, healthcare utilization, and reduced quality of life in middle-aged and older adults, underscoring the urgent need to identify high-risk individuals and develop more precise preventive strategies.

Consequently, CMM may better capture the cumulative consequences of long-standing metabolic dysregulation than a single abnormal measurement obtained at one time point. Existing studies have frequently relied on baseline static indicators for risk assessment, which may overlook the persistence and dynamic evolution of metabolic exposure, thereby limiting the early identification of high-risk individuals (7–9). By contrast, cumulative exposure metrics and change-pattern analyses provide a more comprehensive characterization of the temporal trajectory of metabolic burden, enabling risk stratification that is more closely aligned with the natural history of disease (10, 11). In this context, the recently proposed cholesterol–HDL-C–glucose (CHG) index, derived from routine laboratory measures, has emerged as an integrated marker of overall lipid and glucose metabolic status (12). Previous studies have shown that higher CHG levels are significantly associated with an increased risk of cardiovascular disease, with the relationship appearing to be near-linear (13–15). Furthermore, Tang et al. (16) reported that the CHG index demonstrated risk discrimination comparable to that of established metabolic indices, such as the triglyceride-glucose (TyG) index, supporting its potential as a simple and scalable tool for risk assessment. Nevertheless, the existing literature has focused largely on the association between CHG and individual cardiovascular outcomes. Longitudinal evidence regarding long-term CHG exposure patterns based on repeated measurements in relation to new-onset CMM remains limited, particularly among middle-aged and older Chinese adults.

Against this background, the present study aimed to examine the association of cumulative CHG exposure and CHG patterns with the risk of new-onset CMM. Using longitudinal data from the China Health and Retirement Longitudinal Study (CHARLS), we focused on two longitudinal exposure indicators, cumulative CHG (cuCHG) and CHG patterns based on two repeated measurements. Our findings may strengthen the longitudinal evidence supporting the use of CHG for CMM risk stratification and offer a more practical framework for identifying high-risk middle-aged and older adults.

## Materials and methods

### Study population and participant selection

CHARLS is a nationally representative prospective cohort established to investigate health and aging outcomes among Chinese adults aged 45 years and older. The baseline survey was conducted in 2011, with subsequent follow-up waves in 2013, 2015, 2018, and 2020. Data were collected through computer-assisted personal interviews. At each wave, standardized questionnaires were administered to obtain and update information on demographic characteristics, lifestyle factors, health conditions, and outcome events. In addition, physical examinations and blood samples were obtained at baseline and again in 2015 for the assessment of biochemical markers (17). The study protocol was approved by the Peking University Institutional Review Board (IRB00001052-11015) and was conducted in accordance with the Declaration of Helsinki. Written informed consent was obtained from all participants, and all data were collected by trained field staff using standardized procedures and instruments.

**Figure 1:**
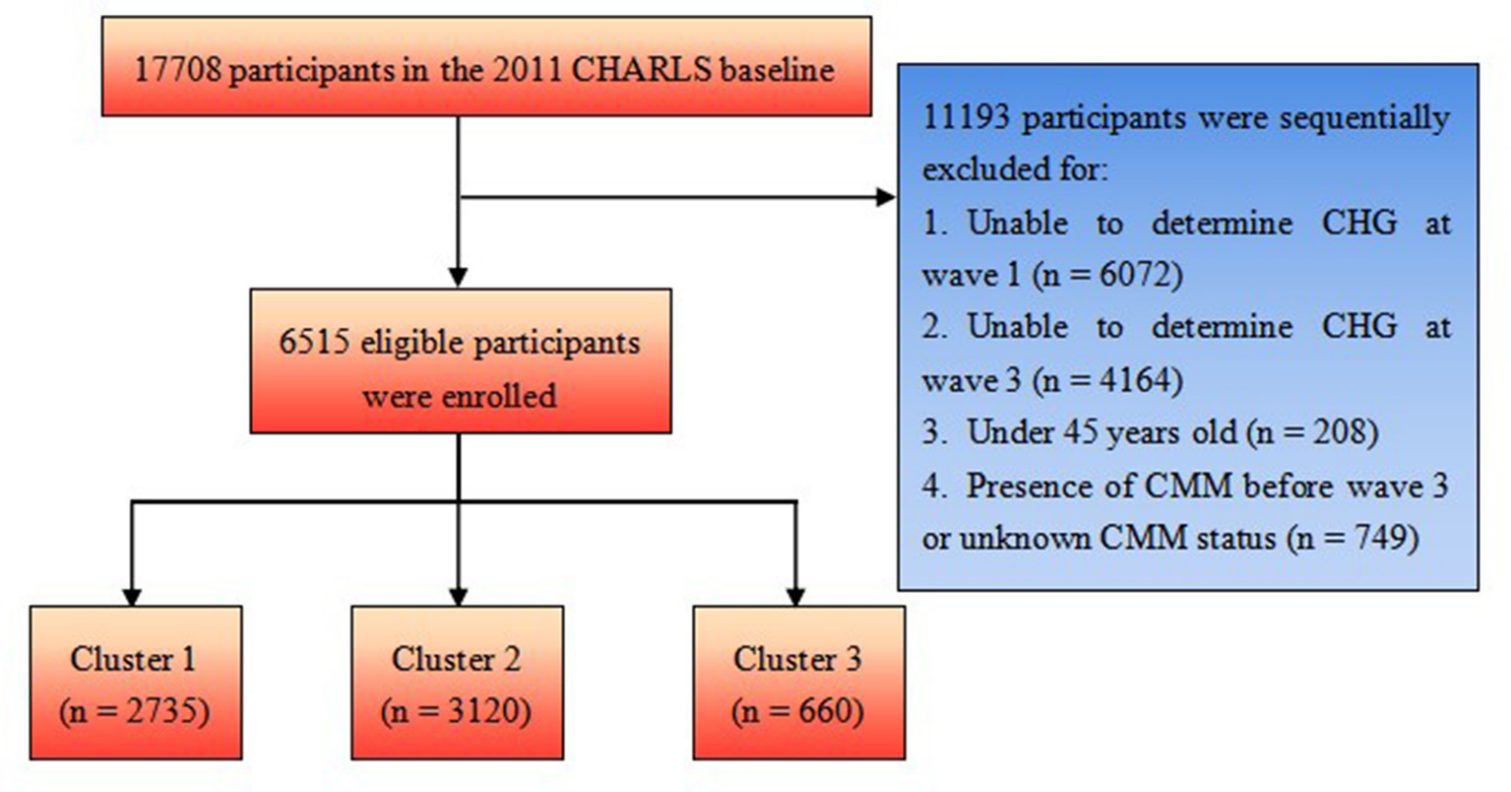
Flowchart of participant selection from CHARLS.

The present study was a secondary longitudinal analysis of the CHARLS cohort. Wave 1 was treated as baseline, and Waves 2 to 5 were used for follow-up. Participants were excluded if they met any of the following criteria: (1) missing data on total cholesterol (TC), fasting blood glucose (FBG), or high-density lipoprotein cholesterol (HDL-C) at Wave 1 or Wave 3; (2) age younger than 45 years; or (3) pre-existing CMM before Wave 3 or inability to ascertain CMM status (Figure 1).

### Definitions

The primary exposures in this study were cuCHG and CHG patterns derived from two repeated measurements obtained in 2012 and 2015. TC, FBG, and HDL-C were extracted from the 2012 and 2015 datasets. The CHG index was calculated as follows: CHG = ln[(TC × FBG)/(2 × HDL-C)]. To capture long-term exposure, cuCHG was calculated using an approach previously applied to cumulative metabolic indices in the CHARLS cohort. Specifically, cumulative exposure from 2012 to 2015 was calculated as the time-weighted average of the two CHG measurements multiplied by the time interval: cuCHG = (CHG2012 + CHG2015) / 2 × time interval (2015–2012). This metric reflects both the average exposure level and the duration of exposure (18, 19).

In addition, the atherogenic index of plasma (AIP) and the TyG index were included as reference indicators. AIP was defined as log_10_(TG/HDL-C) (20), and TyG as ln(TG × FBG/2) (21). These indices were used to compare the discriminative capacity and predictive performance of CHG and its related exposure metrics.

### Outcomes

In the present study, diabetes, stroke, and heart disease were regarded as the three core conditions constituting CMM. CMM was defined as the presence of two or more of these conditions in the same participant during follow-up. To preserve the temporal sequence of event ascertainment, participants with pre-existing CMM at baseline were excluded. Accordingly, the population included in the event analysis comprised participants who had either no cardiometabolic disease or only one of the three conditions at baseline.

Diabetes was identified on the basis of self-reported physician diagnosis, use of glucose-lowering medication, FBG of at least 126 mg/dL, or glycated hemoglobin (HbA1c) of at least 6.5%. Stroke was identified by self-reported physician diagnosis or receipt of stroke-related treatment. Heart disease was defined on the basis of self-reported physician diagnosis of heart attack, coronary heart disease, angina, congestive heart failure, or other heart problems, or the use of cardiovascular medication.

Among participants with 0 or 1 condition at baseline, the occurrence of a second distinct cardiometabolic condition during follow-up was defined as a CMM event. Event ascertainment was based on physician diagnosis, medication use, or relevant clinical measurements. In other words, a CMM event corresponded to the first transition from a non-multimorbid state, defined as 0 or 1 condition, to a multimorbid state, defined as at least 2 conditions. Time to CMM onset was defined as the interval from the start of risk follow-up to the first confirmation of the second distinct condition. In wave-based follow-up data, the event was typically assumed to have occurred between the most recent event-free visit and the first visit at which the event was confirmed.

### Covariates

Information on covariates was collected at baseline, and all multivariable models were adjusted accordingly. The covariates considered in this study included demographic and socioeconomic factors, namely age, sex, education level, residence, and marital status; lifestyle factors, including smoking status, drinking status, and body mass index (BMI); and clinical comorbidity- and treatment-related factors, including hypertension, hypertension treatment, dyslipidemia, dyslipidemia treatment, and nephropathy. To ensure the stability of model estimates, multicollinearity was assessed using variance inflation factors before these covariates were entered into the analyses. All variance inflation factors were below 5 (Supplementary Table S1), indicating no substantial multicollinearity.

### Statistical analysis

Continuous variables are presented as mean ± standard deviation (SD) or median with interquartile range, as appropriate according to their distribution. Categorical variables are presented as counts and percentages. Between-group comparisons were performed using one-way analysis of variance, the Kruskal–Wallis test, or the chi-squared test, as appropriate. To characterize changes in CHG levels between 2012 and 2015, k-means clustering was applied using individual CHG measurements from 2012 and 2015 as input features. This approach was used to identify heterogeneity in both absolute CHG levels and the magnitude of change over time. The optimal number of clusters was determined using the elbow method, based on joint consideration of the within-cluster sum of squares across different cluster solutions, model parsimony, and between-cluster separability. Participants were subsequently classified into distinct CHG pattern groups for further risk analyses.

The primary analyses were conducted using Cox proportional hazards models to evaluate the associations of cuCHG and CHG pattern groups with the risk of new-onset CMM. Results are reported as hazard ratios (HRs) with 95% confidence intervals (CIs). Continuous exposure variables were entered into the models per 1-unit increment, and cuCHG was also analyzed in quartiles, with the lowest quartile serving as the reference group. For trend analyses of ordinal categorical variables, the median value within each category was entered into the model as a continuous variable to derive the P for trend. Three sequential models were constructed: Model 1 was unadjusted; Model 2 was adjusted for age, sex, education level, residence, marital status, smoking status, and drinking status; and Model 3 was further adjusted for BMI, hypertension, hypertension treatment, dyslipidemia, dyslipidemia treatment, and nephropathy. The proportional hazards assumption was assessed using graphical diagnostics and Schoenfeld residuals.

To examine potential non-linear associations between cuCHG and the risk of new-onset CMM, restricted cubic spline (RCS) models were fitted to characterize the dose–response relationship and test for non-linearity. Where evidence of a non-linear association was observed, a two-piecewise Cox proportional hazards model was applied to explore potential threshold effects. Model fit for the piecewise model was compared with that of the single linear model using a likelihood ratio test. This approach allowed identification of a potential inflection point and estimation of effect sizes on either side of the threshold. To explore potential effect modification, the primary analyses were repeated across subgroups defined by age, sex, residence, smoking status, drinking status, BMI, hypertension, dyslipidemia, and nephropathy. All subgroup analyses retained the covariates included in the main model, and interaction terms between the exposure and subgroup variable were introduced to test for interaction; corresponding *P* values for interaction were reported. In addition, receiver operating characteristic (ROC) curve analyses were performed to compare the discriminative ability and predictive performance of CHG-related exposure metrics with those of the reference indicators, namely AIP and TyG, for new-onset CMM. The area under the curve (AUC) was calculated accordingly. For time-to-event outcomes, time-dependent ROC analyses were further conducted to assess the predictive performance of each indicator at prespecified time points and to compare their AUCs. To assess the robustness of the findings, three sensitivity analyses were performed. First, the primary analyses were repeated after excluding participants with missing covariates to evaluate the potential influence of sample selection bias. Second, to reduce the potential influence of extreme values on model estimates, participants with cuCHG values beyond the mean ± 3 SD were excluded. In addition, because BMI was the only continuous covariate, participants with BMI values below the 1st percentile or above the 99th percentile were also excluded before reanalysis. Third, Model 3 was additionally adjusted for biochemical markers, including uric acid, C-reactive protein, low-density lipoprotein cholesterol, blood urea nitrogen, and HbA1c, to further assess the potential influence of residual confounding.

To address missing covariate data, multiple imputation by chained equations under fully conditional specification was performed. Twenty imputed datasets were generated with 10 iterations per dataset, using variable-specific imputation models according to variable type. The imputation model included all covariates used in the main analyses, together with the exposure and outcome variables. Estimates from the imputed datasets were combined using Rubin's rules. To assess the adequacy of the imputations, we examined trace plots of the mean and standard deviation across iterations and compared the distributions of observed and imputed values. Patterns of missing covariates are shown in Supplementary Table S2. All tests were two-sided, and a *P* value < 0.05 was considered statistically significant. All statistical analyses were performed using R software, version 4.4.1.

## Results

### Baseline characteristics

A total of 17,708 participants were enrolled in the 2011 baseline CHARLS cohort and screened sequentially according to the prespecified inclusion and exclusion criteria. Participants were excluded for the following reasons: inability to calculate the CHG index at Wave 1 (*n* = 6,072) or Wave 3 (*n* = 4,164), age below 45 years (*n* = 208), and prevalent CMM before Wave 3 or indeterminate CMM status (*n* = 749). The final analysis included 6,515 participants who met the eligibility criteria. Based on K-means clustering of the two repeated CHG measurements, participants were classified into three groups: Cluster 1 (*n* = 2,735), Cluster 2 (*n* = 3,120), and Cluster 3 (*n* = 660) (Figure 1).

Over a median follow-up of 9 years, 593 participants (9.1%) developed new-onset CMM. Table 1 summarizes the baseline characteristics of the study population according to CHG pattern group. Cluster 1 represented the low-level group, with CHG declining from 5.0 ± 0.2 in 2012 to 4.9 ± 0.2 in 2015. Cluster 2 represented the moderate-level group, with CHG decreasing from 5.4 ± 0.2 to 5.3 ± 0.2. Cluster 3 had the highest CHG levels, declining from 6.1 ± 0.5 to 5.8 ± 0.4. Overall, CHG levels showed a modest decline between 2012 and 2015 across all three groups, while clear between-group separation was maintained. CHG measured in 2012 and 2015, as well as cuCHG, differed significantly across the three clusters (all *P* < 0.001), with progressively higher levels from Cluster 1 to Cluster 3. In addition, the three groups differed in sex distribution, place of residence, and lifestyle characteristics. Compared with Cluster 1, participants in the higher CHG pattern groups had higher BMI, a greater prevalence of hypertension, diabetes, and dyslipidemia, and more frequent use of related medications. They also showed a less favorable biochemical profile, characterized primarily by more pronounced disturbances in glucose and lipid metabolism.

**Table 1:** Baseline characteristics of participants.

Characteristics	Cluster				*P value*
	Total (*n* = 6,515)	Cluster 1 (*n* = 2,735)	Cluster 2 (*n* = 3,120)	Cluster 3 (*n* = 660)	
Age, years	58.4 ± 8.7	58.2 ± 8.8	58.6 ± 8.6	58.5 ± 8.1	0.169
<60, *n* (%)	3,779 (58.0)	1,601 (58.5)	1,797 (57.6)	381 (57.7)	
≥60, *n* (%)	2,736 (42.0)	1,134 (41.5)	1,323 (42.4)	279 (42.3)	
Men, *n* (%)	3,041 (46.7)	1,344 (49.1)	1,411 (45.2)	286 (43.3)	0.002
Residence, *n* (%)					<0.001
Rural	4,333 (66.5)	1,977 (72.3)	1,976 (63.3)	380 (57.6)	
Urban	2,182 (33.5)	758 (27.7)	1,144 (36.7)	280 (42.4)	
Marital, *n* (%)					0.613
Unmarried	975 (15.0)	422 (15.4)	460 (14.7)	93 (14.1)	
Married	5,540 (85.0)	2,313 (84.6)	2,660 (85.3)	567 (85.9)	
Education level, *n* (%)					0.076
No formal education	1,873 (28.7)	813 (29.7)	887 (28.4)	173 (26.2)	
Primary school	2,717 (41.7)	1,161 (42.4)	1,282 (41.1)	274 (41.5)	
Middle school or above	1,925 (29.5)	761 (27.8)	951 (30.5)	213 (32.3)	
BMI, kg/m^2^	23.2 (20.9, 25.7)	21.8 (20.0, 24.1)	23.9 (21.7, 26.4)	25.3 (23.1, 27.7)	<0.001
<25, *n* (%)	4,500 (69.1)	2,240 (81.9)	1,953 (62.6)	307 (46.5)	
≥25, *n* (%)	2,015 (30.9)	495 (18.1)	1,167 (37.4)	353 (53.5)	
Smoking status, *n* (%)					<0.001
No	4,568 (70.1)	1,844 (67.4)	2,245 (72)	479 (72.6)	
Yes	1,947 (29.9)	891 (32.6)	875 (28)	181 (27.4)	
Drinking status, *n* (%)					<0.001
No	4,295 (65.9)	1,705 (62.3)	2,119 (67.9)	471 (71.4)	
Yes	2,220 (34.1)	1,030 (37.7)	1,001 (32.1)	189 (28.6)	
Hypertension, *n* (%)	2,706 (41.5)	922 (33.7)	1,406 (45.1)	378 (57.3)	<0.001
Hypertension treatment, *n* (%)	1,146 (17.6)	336 (12.3)	607 (19.5)	203 (30.8)	<0.001
Dyslipidemia, *n* (%)	632 (9.7)	153 (5.6)	342 (11)	137 (20.8)	<0.001
Dyslipidemia treatment, *n* (%)	292 (4.5)	71 (2.6)	154 (4.9)	67 (10.2)	<0.001
Nephropathy, *n* (%)	401 (6.2)	159 (5.8)	194 (6.2)	48 (7.3)	0.368
Diabetes, *n* (%)	1,434 (22.0)	234 (8.6)	710 (22.8)	490 (74.2)	<0.001
Stroke, *n* (%)	127 (1.9)	52 (1.9)	63 (2.0)	12 (1.8)	0.918
Heart disease, *n* (%)	920 (14.1)	430 (15.7)	446 (14.3)	44 (6.7)	<0.001
New onset CMM, *n* (%)	593 (9.1)	173 (6.3)	292 (9.4)	128 (19.4)	<0.001
HDL-C, mg/dl	51.4 ± 15.3	60.6 ± 15.1	46.5 ± 11.1	36.8 ± 11.2	<0.001
LDL-C, mg/dl	116.6 ± 33.2	112.6 ± 32.0	119.4 ± 32.7	119.6 ± 38.7	<0.001
FBG, mg/dl	107.9 ± 32.7	97.3 ± 13.8	106.8 ± 18.2	157.1 ± 73.1	<0.001
HbA1c, %	5.2 ± 0.8	5.1 ± 0.4	5.2 ± 0.5	6.2 ± 1.7	<0.001
TC, mg/dl	193.1 ± 38.4	179.4 ± 32.6	199.3 ± 35.8	220.5 ± 48.7	<0.001
TG, mg/dl	104.4 (74.3, 151.8)	79.7 (62.0, 106.2)	120.4 (88.5, 166.4)	200.5 (137.2, 316.2)	<0.001
BUN, mg/dL	15.2 ± 4.3	15.3 ± 4.4	15.1 ± 4.1	15.1 ± 3.9	0.053
Creatinine, mg/dL	1.0 (0.6, 2.2)	1.0 (0.5, 2.2)	1.1 (0.6, 2.2)	1.2 (0.7, 2.7)	<0.001
Uric acid, mg/dL	4.5 ± 1.1	4.4 ± 1.1	4.5 ± 1.1	4.7 ± 1.1	<0.001
CHG_2012_	5.3 ± 0.4	5.0 ± 0.2	5.4 ± 0.2	6.1 ± 0.5	<0.001
CHG_2015_	5.2 ± 0.4	4.9 ± 0.2	5.3 ± 0.2	5.8 ± 0.4	<0.001
cuCHG	15.7 ± 1.1	14.8 ± 0.5	16.1 ± 0.4	17.9 ± 0.9	<0.001

BMI, body mass index; BUN, blood urea nitrogen; CMM, Cardiometabolic Multimorbidity; FBG, fasting blood glucose; HbA1c, hemoglobin A1c; HDL-C, high density lipoprotein cholesterol; LDL-C, low-density lipoprotein cholesterol; TC, total cholesterol; TG, triglycerides.

**Figure 2:**
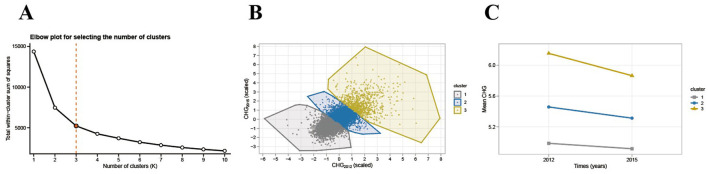
K-means clustering analysis of CHG patterns based on two repeated measurements between 2012 and 2015. **(A)** Elbow plot showing the selection of the optimal number of clusters (K = 3). **(B)** Scatter plot of participants according to standardized CHG values in 2012 and 2015, showing the three identified clusters. **(C)** Mean CHG values in 2012 and 2015 for each cluster, illustrating distinct low-, moderate-, and high-level CHG patterns, with a slight overall decline over time.

Figure 2 shows the results of the k-means clustering analysis based on CHG values measured in 2012 and 2015, together with the procedure used to determine the optimal number of clusters. The elbow method (Figure 2A) showed that the within-cluster sum of squares declined sharply up to k = 3, suggesting that a three-cluster solution provided a reasonable balance between capturing heterogeneity and preserving model parsimony. Accordingly, k = 3 was selected as the optimal number of clusters. As shown in Figure 2B, the three clusters were clearly separated in the two-dimensional space defined by standardized CHG values in 2012 and 2015. Figure 2C further shows that all three clusters exhibited declining CHG levels between 2012 and 2015 while maintaining clear hierarchical separation. These CHG pattern groups were subsequently used to assess their associations with the risk of new-onset CMM.

### Cox regression analysis

Table 2 presents three Cox proportional hazards models evaluating the associations of cuCHG and CHG pattern groups with the risk of new-onset CMM. In Model 1, the unadjusted model, each 1-unit increase in cuCHG was significantly associated with a 35% higher risk of new-onset CMM (HR 1.35, 95% CI 1.27–1.43, *P* < 0.001). This association remained essentially unchanged after adjustment for demographic and lifestyle factors in Model 2 (HR 1.36, 95% CI 1.27–1.44, *P* < 0.001). In the fully adjusted Model 3, cuCHG remained independently associated with new-onset CMM, with each 1-unit increase corresponding to a 23% higher risk (HR 1.23, 95% CI 1.15–1.32, *P* < 0.001). In the quartile analysis, participants in the highest cuCHG quartile (Q4) had a 78% higher risk of new-onset CMM than those in the lowest quartile (Q1) in Model 3 (HR 1.78, 95% CI 1.39–2.29, *P* < 0.001), with a significant dose-response trend across quartiles (*P* for trend < 0.001).

**Table 2:** Associations of cuCHG and CHG pattern groups with the risk of new-onset CMM.

Character	Event, *n* (%)	Model 1		Model 2		Model 3	
		HR (95% CI)	*P* value	HR (95% CI)	*P* value	HR (95% CI)	*P* value
cuCHG	593 (9.1)	1.35 (1.27–1.43)	<0.001	1.36 (1.27–1.44)	<0.001	1.23 (1.15–1.32)	<0.001
Q1	104 (6)	Reference	—	Reference	—	Reference	—
Q2	119 (7.1)	1.15 (0.88–1.50)	0.298	1.13 (0.87–1.47)	0.357	1.05 (0.80–1.36)	0.741
Q3	150 (9.1)	1.40 (1.09–1.80)	0.008	1.34 (1.04–1.72)	0.022	1.13 (0.87–1.46)	0.356
Q4	220 (15.2)	2.51 (1.98–3.16)	<0.001	2.45 (1.93–3.10)	<0.001	1.78 (1.39–2.29)	<0.001
*P* trend			<0.001		<0.001		<0.001
CHG pattern groups							
Cluster 1	173 (6.3)	Reference	—	Reference	—	Reference	—
Cluster 2	292 (9.4)	1.43 (1.19–1.73)	<0.001	1.38 (1.14–1.67)	0.001	1.16 (0.95–1.41)	0.136
Cluster 3	128 (19.4)	2.99 (2.38–3.75)	<0.001	2.94 (2.33–3.71)	<0.001	2.05 (1.61–2.61)	<0.001
*P* trend			<0.001		<0.001		<0.001

Model 1: unadjusted; Model 2: adjusted for age, sex, education, residence, marriage, smoking status, and drinking status; Model 3: fully adjusted for age, sex, education, residence, marriage, smoking status, drinking status, BMI, hypertension, hypertension treatment, dyslipidemia, dyslipidemia treatment, and nephropathy.

BMI, body mass index; CHG, cholesterol, high-density lipoprotein, and glucose; CI, confidence interval; CMM, cardiometabolic multimorbidity; HR, hazard ratio.

In the CHG pattern group analysis, the moderate-level group (Cluster 2) showed a higher risk of new-onset CMM than the low-level group (Cluster 1) in Models 1 and 2. However, this association was attenuated and no longer statistically significant after full adjustment in Model 3 (HR 1.16, 95% CI 0.95–1.41, *P* = 0.136). By contrast, the high-level group (Cluster 3) consistently showed a significantly higher risk of new-onset CMM across all three models. In Model 3, the risk was 105% higher than that in Cluster 1 (HR 2.05, 95% CI 1.61–2.61, *P* < 0.001). A significant increasing trend in risk was observed across CHG pattern groups (*P* for trend < 0.001).

### RCS and threshold effect

**Figure 3:**
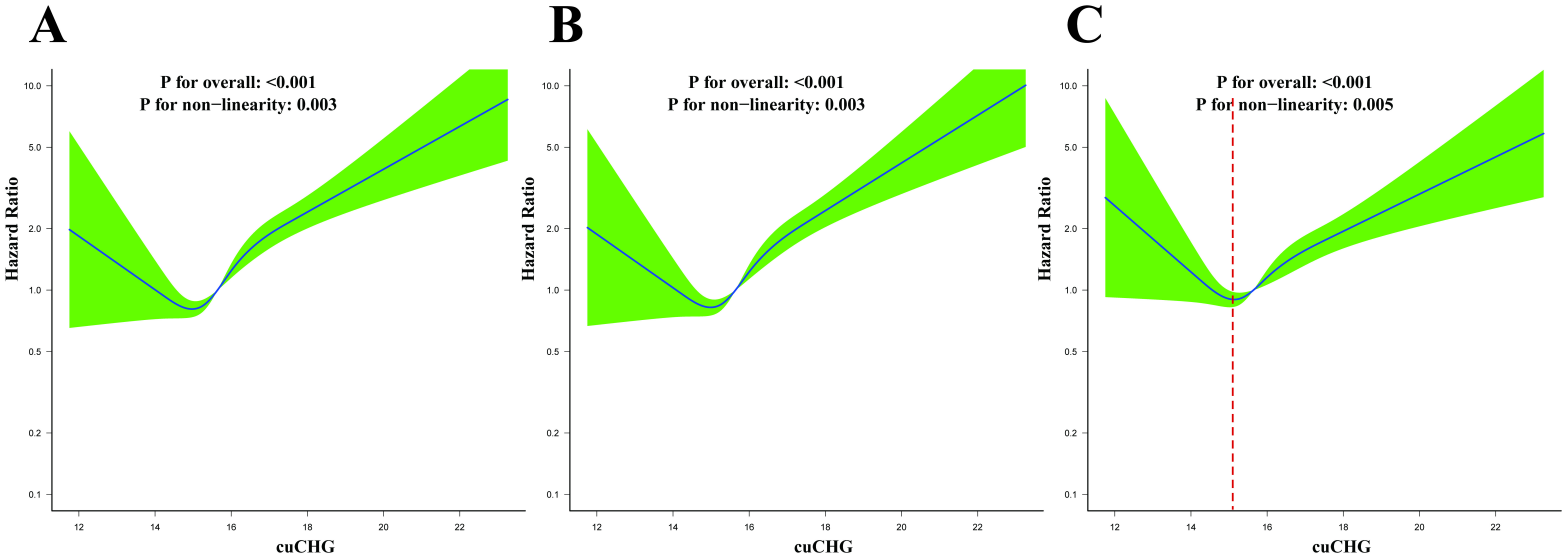
RCS analyses of the association between cuCHG and new-onset CMM. **(A)** Model 1, unadjusted. **(B)** Model 2, adjusted for age, sex, education level, residence, marital status, smoking status, and drinking status. **(C)** Model 3, further adjusted for BMI, hypertension, hypertension treatment, dyslipidemia, dyslipidemia treatment, and nephropathy. The blue solid lines represent HRs, and the green shaded areas indicate 95% CIs. A significant non-linear association between cuCHG and new-onset cardiometabolic multimorbidity was observed in all three models. The red dashed line in panel C marks the statistical inflection point identified by the two-piecewise Cox proportional hazards model.

To further evaluate the potential non-linear association between cuCHG and the risk of new-onset CMM, RCS analyses (Figure 3) and a two-piecewise Cox proportional hazards model (Supplementary Table S3) were performed. Across progressively adjusted models, the RCS curves showed a significant non-linear association between cuCHG and the risk of new-onset CMM (*P* < 0.001; P for non-linearity = 0.005 in Model 3). Using the two-piecewise Cox proportional hazards model, a statistical inflection point for cuCHG was identified at 14.92. In the fully adjusted model, the association between cuCHG and new-onset CMM was not statistically significant when cuCHG was below 14.92 (HR 0.64, 95% CI 0.39–1.06, *P* = 0.084). However, when cuCHG exceeded 14.92, it was significantly and positively associated with the risk of new-onset CMM (HR 1.31, 95% CI 1.19–1.44, *P* < 0.001). Similar patterns were observed in Models 1 and 2. In addition, log-likelihood ratio tests indicated that the two-piecewise Cox proportional hazards model provided a better fit than the single linear model.

### Subgroup and interaction analyses

**Figure 4:**
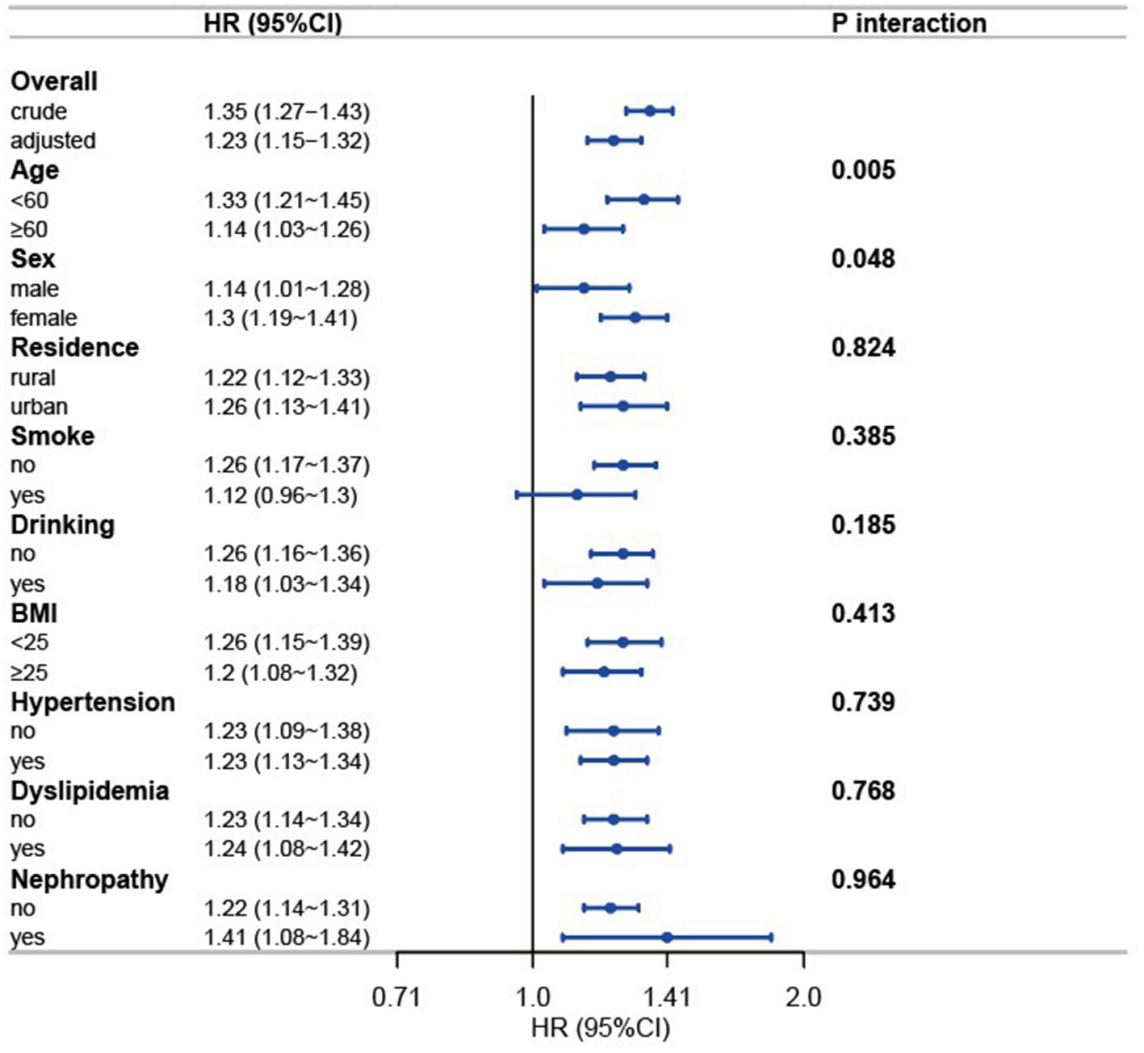
Forest plot of subgroup analyses of the association between cuCHG and new-onset CMM. HRs and 95% CIs are shown for the overall population and predefined subgroups. The adjusted model included demographic, lifestyle, and clinical covariates, except for the corresponding stratification variable. Significant interactions were identified for age and sex.

Figure 4 presents the subgroup analysis of cuCHG. The positive association between cuCHG and new-onset CMM remained consistent across most subgroups. Statistically significant interactions were observed for age (*P* for interaction = 0.005) and sex (*P* for interaction = 0.048). No significant interactions were identified for residence, smoking status, drinking status, BMI, hypertension, dyslipidemia, or nephropathy.

In the subgroup analysis of CHG pattern groups (Table 3), Cluster 3 was significantly associated with a higher risk of new-onset CMM in most subgroups, using Cluster 1 as the reference, and a significant dose-response trend was observed across CHG pattern groups. A significant interaction was observed for age (*P* for interaction = 0.003), whereas no significant interactions were detected in the other subgroups. Overall, these findings suggest that the association between CHG pattern groups and the risk of new-onset CMM was broadly consistent across subgroups.

**Table 3:** Subgroup analysis of the association between CHG pattern groups and the risk of new-onset CMM.

Character	Cluster 1	Cluster 2	Cluster 3	*P* trend	*P* interaction
Age					0.003
<60	Ref.	1.66 (1.22–2.28)	2.67 (1.82–3.91)	<0.001	
≥60	Ref.	0.9 (0.7–1.16)	1.74 (1.26–2.4)	0.011	
Sex					0.094
Male	Ref.	0.96 (0.72–1.28)	1.69 (1.16–2.48)	0.037	
Female	Ref.	1.39 (1.06–1.82)	2.44 (1.76–3.37)	<0.001	
Residence					0.448
Rural	Ref.	1.27 (1–1.61)	2.12 (1.55–2.9)	<0.001	
Urban	Ref.	1.03 (0.73–1.45)	1.98 (1.33–2.94)	0.001	
Smoke					0.755
No	Ref.	1.19 (0.95–1.5)	2.22 (1.68–2.94)	<0.001	
Yes	Ref.	1.1 (0.76–1.61)	1.54 (0.93–2.55)	0.129	
Drinking					0.235
No	Ref.	1.28 (1–1.62)	2.17 (1.62–2.9)	<0.001	
Yes	Ref.	0.96 (0.68–1.34)	2.05 (1.3–3.25)	0.027	
BMI					
<25	Ref.	1.18 (0.92–1.5)	2.35 (1.68–3.29)	<0.001	0.410
≥25	Ref.	1.06 (0.77–1.48)	1.73 (1.2–2.5)	0.002	
Hypertension					0.282
No	Ref.	1.35 (1.01–1.82)	2.22 (1.47–3.36)	<0.001	
Yes	Ref.	1.02 (0.79–1.32)	1.88 (1.39–2.54)	<0.001	
Dyslipidemia					0.686
No	Ref.	1.14 (0.92–1.41)	2.14 (1.62–2.83)	<0.001	
Yes	Ref.	1.07 (0.65–1.75)	1.75 (1.03–2.99)	0.020	
Nephropathy					0.871
No	Ref.	1.16 (0.94–1.42)	2.02 (1.56–2.61)	<0.001	
Yes	Ref.	1.39 (0.66–2.92)	3.03 (1.28–7.18)	0.015	

The model was adjusted for age, sex, education, residence, marriage, smoking status, drinking status, BMI, hypertension, hypertension treatment, dyslipidemia, dyslipidemia treatment, and nephropathy.

BMI, body mass index; CHG, cholesterol, high-density lipoprotein, and glucose; CI, confidence interval; CMM, cardiometabolic multimorbidity; HR, hazard ratio.

### Predictive performance

**Figure 5:**
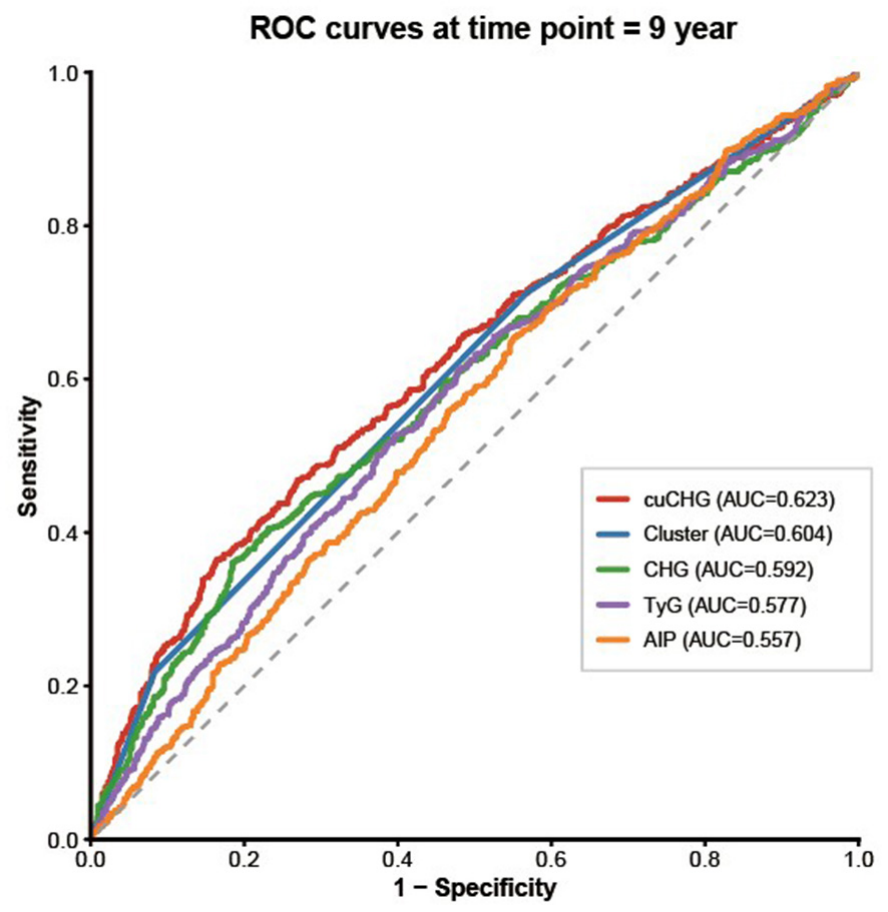
Time-dependent ROC curves for related indicators in predicting new-onset CMM at 9 years. The predictive performance of cuCHG, CHG pattern groups, baseline CHG, TyG, and AIP was compared by time-dependent ROC analysis. cuCHG showed the highest discriminative ability, followed by CHG pattern groups and baseline CHG.

Figure 5 presents the ROC curves for the various indicators used to predict new-onset CMM at the 9-year follow-up. Among these indicators, cuCHG showed the greatest discriminative ability, with an AUC of 0.623. This was followed by CHG pattern groups (AUC = 0.604) and baseline CHG (AUC = 0.592). Baseline TyG (AUC = 0.577) and baseline AIP (AUC = 0.557) showed weaker predictive performance. Although the overall AUC values were modest, cuCHG and CHG pattern groups demonstrated comparatively better discriminative ability than the other indicators.

### Sensitivity analyses

To assess the robustness of the primary findings, three sensitivity analyses were performed to address potential sample selection bias, the influence of outliers, and residual confounding by additional biochemical variables. First, after excluding participants with missing covariates, both cuCHG and CHG pattern groups remained significantly and positively associated with the risk of new-onset CMM, with a clear dose-response pattern (Supplementary Table S4). Second, after excluding participants with extreme cuCHG and BMI values, the associations of cuCHG and CHG pattern groups with new-onset CMM remained robust, with slightly higher overall HR estimates (Supplementary Table S5). Third, after further adjustment for additional biochemical markers, including uric acid, C-reactive protein, low-density lipoprotein cholesterol, blood urea nitrogen, and HbA1c, the results remained materially unchanged (Supplementary Table S6). Taken together, these sensitivity analyses were consistent with the primary analyses and further supported the robustness of the findings.

## Discussion

The present study used longitudinal data from CHARLS to systematically examine the associations of cumulative and pattern-based CHG exposure characteristics with the risk of new-onset CMM in middle-aged and older adults. Several key findings emerged. First, higher cuCHG levels, analyzed either as a continuous variable or in quartiles, were significantly associated with an increased risk of new-onset CMM, with a clear dose–response pattern. Second, CHG patterns based on two repeated measurements showed that individuals in the persistently high CHG pattern group were at significantly greater risk of developing new-onset CMM than those in the low CHG pattern group. By contrast, the association for the intermediate CHG pattern group was attenuated after full adjustment, suggesting that sustained high exposure may be of greater clinical relevance. Third, RCS and two-piecewise Cox proportional hazards models indicated a non-linear association between cuCHG and the risk of new-onset CMM, with a statistical inflection point at cuCHG = 14.92. In addition, the observed associations were broadly consistent across most subgroups, with evidence of interaction only by age and sex. Finally, cuCHG and CHG pattern groups demonstrated better discriminative ability than baseline CHG, TyG, and AIP. The consistency of the findings across multiple sensitivity analyses further supports the robustness of the results.

The principal contribution of the present study lies in extending the characterization of CHG-related risk, rather than simply reaffirming previously reported associations. Recent studies have begun to expand the application of CHG beyond its conventional role as a general metabolic risk marker, thereby supporting its use in more specific clinical contexts. For example, Song et al. (22) showed that CHG was an independent predictor of adverse outcomes and provided additional value for risk reclassification in two large prospective cohorts of patients with myocardial infarction. Likewise, Liu et al. (23) reported a significant association between CHG and CMM in both a nationwide prospective cohort study and a multi-community cross-sectional study. However, most existing evidence has relied primarily on baseline CHG levels to assess outcome risk, with limited attention to long-term cumulative exposure or broader longitudinal patterns based on repeated measurements (24–28). In contrast, the present study focused specifically on new-onset CMM and incorporated both cuCHG and CHG patterns based on two repeated measurements. This approach captures long-term metabolic burden from the complementary perspectives of cumulative exposure and temporal evolution. It also reflects the real-world process through which metabolic abnormalities accumulate over time, promoting progression from a single cardiometabolic condition to multimorbidity in middle-aged and older adults. Moreover, the non-linear association observed in the present analyses suggests that the relationship between cuCHG and new-onset CMM may not increase uniformly across the exposure range. Rather, the positive association became more evident when cuCHG exceeded approximately 14.92, whereas no statistically significant association was observed below this point. This value should be interpreted as a statistical inflection point identified within this dataset rather than a clinical cut-off. Compared with a single baseline CHG measurement, cumulative CHG exposure and CHG patterns based on repeated measurements may provide additional information for CMM risk stratification, consistent with their better, although still modest, discriminative performance relative to baseline CHG, TyG, and AIP.

An additional point that merits consideration is that CHG levels declined modestly between 2012 and 2015 across all three CHG pattern groups. This pattern may have several explanations. Participants in the higher-CHG groups had a greater burden of diabetes, dyslipidemia, and related treatment use at baseline, which may have increased the likelihood of treatment intensification and lifestyle modification during follow-up. In addition, because CHG was assessed at only two time points, part of the observed decline may reflect regression to the mean and within-person biological variation. Importantly, however, the three groups remained clearly separated over time, suggesting that the pattern classification still captured meaningful relative differences in long-term metabolic burden.

From a mechanistic perspective, the associations observed in the present study are biologically plausible, although direct mechanistic evidence for the CHG index itself remains limited. Because CHG integrates TC, FBG, and HDL-C, it may reflect broader disturbances in glucose and lipid metabolism and, to some extent, the long-term metabolic burden associated with insulin resistance. Emerging studies have linked higher CHG levels to diabetic microvascular complications, cardiometabolic multimorbidity, and adverse cardiovascular outcomes, supporting its role as an integrated metabolic marker rather than a direct mechanistic mediator (12, 22, 29). Persistent hyperglycaemia and lipid dysregulation may in turn promote atherosclerotic progression through several interacting pathways, including disruption of endothelial homeostasis, sustained inflammatory activation, increased oxidative stress, and the development of a prothrombotic milieu (30–35). Importantly, these pathological processes are unlikely to be confined to a single organ-specific outcome, but may instead contribute simultaneously to the accumulation of risk for diabetes, heart disease, and stroke (36), thereby facilitating progression from a single cardiometabolic condition to CMM. Evidence from studies of insulin resistance-related surrogate markers, such as the TyG index, also supports a similar mechanistic pathway, whereby associations with cardio-cerebrovascular risk may be partly mediated through endothelial dysfunction, inflammation, and accelerated atherosclerosis (37–40). Furthermore, the non-linear association and statistical inflection point observed between cuCHG exposure and CMM risk in our study raise the possibility that the effect of this exposure on CMM progression may be stage-dependent. In other words, once long-term metabolic burden exceeds a certain level, its contribution to multisystem damage may become more pronounced. This interpretation, however, remains speculative and requires further investigation through longitudinal mechanistic studies and mediation analyses.

From clinical and public health perspectives, the findings of this study suggest that CHG-related metrics, particularly cuCHG and CHG pattern groups, may offer a simple and consistent means of identifying the risk of new-onset CMM at an early stage in middle-aged and older adults. Because CHG is derived from routinely measured laboratory parameters, including TC, FBG, and HDL-C, it is relatively inexpensive and widely accessible. This makes it well suited to serial monitoring in community follow-up programmes and in primary care-based chronic disease management. Compared with reliance on a single baseline measurement, our findings suggest that incorporating cumulative exposure and repeated-measure pattern information may help identify individuals with persistently elevated metabolic burden, who may represent a priority group for preventing progression from a single cardiometabolic condition to CMM. Nevertheless, although cuCHG and CHG pattern groups showed better discrimination than baseline CHG, TyG, and AIP, the overall AUC values remained modest. This suggests that CHG-related metrics may be more useful as a complement to existing risk assessment frameworks than as stand-alone diagnostic tools. Future studies should therefore explore whether integrating cumulative and pattern-based CHG metrics with conventional risk factors, clinical history, and other biomarkers can improve identification of individuals at risk of CMM progression and enhance clinical utility.

The present study has several strengths. First, it evaluated the associations between cumulative and pattern-based CHG exposure characteristics and the risk of new-onset CMM in middle-aged and older adults using the nationally representative longitudinal CHARLS cohort, thereby enhancing the population-level relevance of the findings. Second, in addition to baseline CHG, we incorporated cuCHG and CHG patterns based on two repeated measurements to provide a more comprehensive characterization of long-term metabolic burden from the complementary perspectives of cumulative exposure and temporal change patterns. We also combined RCS analyses, two-piecewise Cox proportional hazards models, subgroup analyses, and multiple sensitivity analyses to provide a relatively comprehensive assessment of the association pattern and the robustness of the findings. Several limitations should nevertheless be acknowledged. First, as this was an observational study, residual confounding and reverse causation cannot be completely excluded, despite adjustment for a wide range of potential confounders and additional biochemical markers in sensitivity analyses. Second, the core components of CMM were identified primarily on the basis of self-reported diagnoses and treatment information, which may have introduced misclassification. Third, CHG trajectory patterns and cuCHG were derived from only two measurements obtained in 2012 and 2015; therefore, these measures reflect broad longitudinal exposure patterns rather than fully capturing more complex temporal fluctuations over time. In addition, caution is warranted when generalizing these findings to younger populations or to populations with different ethnic or regional backgrounds, as the study population comprised middle-aged and older Chinese adults. Despite these limitations, the direction of the associations remained consistent across different modeling strategies and multiple sensitivity analyses, which supports the robustness of the findings.

## Conclusion

In conclusion, this longitudinal analysis of the CHARLS cohort among middle-aged and older adults showed that higher cuCHG levels and a persistently high CHG pattern group were significantly associated with an increased risk of new-onset CMM. The association between cuCHG and CMM risk appeared to be non-linear, with evidence of a statistical inflection point. Compared with a single baseline CHG measurement, cumulative CHG exposure and CHG patterns based on repeated measurements may provide additional information for CMM risk stratification. Nevertheless, further external validation in other populations and mechanistic studies are needed to clarify the biological basis and clinical utility of this approach.

## Data Availability

The data used in this study are available from the CHARLS repository (https://charls.pku.edu.cn). Access to the data can be obtained via the official CHARLS website upon reasonable request and completion of the data use agreement. The analytic code used in this study is available from the corresponding author upon request.
