# Supplementary material for: Cumulative exposure and patterns of the cholesterol–HDL-C–glucose index and risk of new-onset cardiometabolic multimorbidity: 9-year longitudinal evidence from CHARLS
Source: Front Nutr. 2026 May 25;13:1835874. doi: 10.3389/fnut.2026.1835874 (PMC13243423; doi:10.3389/fnut.2026.1835874)
Supplement: Supplementary file 1 [file Supplementary_file_1.DOC]

**Table S1. Multicollinearity analysis of covariates included in the multivariable models**

| **Variable** | **GVIF** | **DF** | **GVIF^(1/(2×DF))** | **Colinearity** |
| --- | --- | --- | --- | --- |
| Age | 1.156 | 1.0 | 1.075 | 0.0 |
| Sex | 1.787 | 1.0 | 1.337 | 0.0 |
| Residence | 1.102 | 1.0 | 1.05 | 0.0 |
| Marital | 1.064 | 1.0 | 1.032 | 0.0 |
| Education | 1.37 | 2.0 | 1.082 | 0.0 |
| BMI | 1.206 | 1.0 | 1.098 | 0.0 |
| Smoke | 1.363 | 1.0 | 1.168 | 0.0 |
| Drink | 1.354 | 1.0 | 1.164 | 0.0 |
| Dyslipidaemia | 1.834 | 1.0 | 1.354 | 0.0 |
| Dyslipidaemia treatment | 1.82 | 1.0 | 1.349 | 0.0 |
| Hypertension | 1.567 | 1.0 | 1.252 | 0.0 |
| Hypertension treatment | 1.636 | 1.0 | 1.279 | 0.0 |
| Nephropathy | 1.034 | 1.0 | 1.017 | 0.0 |

Abbreviation: GVIF, generalized variance inflation factor; DF, degrees of freedom

**Table S2. Distribution of missing variables**

| **Variables** | **Number of Missing** | **Missing proportion** |
| --- | --- | --- |
| Age | 11 | 0.17% |
| Smoking status | 2 | 0.03% |
| Drinking status | 1 | 0.02% |
| BMI | 15 | 0.23% |

Abbreviation: BMI, body mass index.

# **Table S3. Threshold effects of the cuCHG on risk of new-onset CMM using a two-piecewise linear regression model**

| **Character** | **Sample,**  **n (%)** | **Model 1** | | **Model 2** | | **Model 3** | |
| --- | --- | --- | --- | --- | --- | --- | --- |
|  |  | **HR (95% CI)** | **P value** | **HR (95% CI)** | **P value** | **HR (95% CI)** | **P value** |
| All | 6482 | 1.35 (1.27-1.43) | < 0.001 | 1.36 (1.27-1.44) | < 0.001 | 1.23 (1.15-1.32) | < 0.001 |
| Fitting by two-piecewise Cox proportional risk model |  |  |  |  |  |  |  |
| Inflection point |  | 14.92 |  | 14.92 |  | 14.92 |  |
| cuCHG < 14.92 | 1410 | 0.58 (0.33-1.01) | 0.054 | 0.72 (0.42-1.24) | 0.238 | 0.64 (0.39-1.06) | 0.084 |
| cuCHG ≥ 14.92 | 5072 | 1.44 (1.32-1.56) | < 0.001 | 1.44 (1.32-1.57) | < 0.001 | 1.31 (1.19-1.44) | < 0.001 |
| P for Log-likelihood ratio |  |  | 0.003 |  | 0.005 |  | 0.009 |

Model 1: unadjusted; Model 2: adjusted for age, sex, education, residence, marriage, smoking status, drinking status; Model 3: fully adjusted for age, sex, education, residence, marriage, smoking status, drinking status, BMI, hypertension, hypertension treatment, dyslipidaemia, dyslipidaemia treatment, and nephropathy. Abbreviation: BMI, body mass index; CHG: Cholesterol, High-Density Lipoprotein, and Glucose; CI, confidence interval; CMM: Cardiometabolic Multimorbidity; HR, hazard ratio.

# **Table S4. Sensitivity analysis after excluding individuals with missing data**

| **Character** | **Event, n (%)** | **Model 1** | | **Model 2** | | **Model 3** | |
| --- | --- | --- | --- | --- | --- | --- | --- |
|  |  | **HR (95% CI)** | **P value** | **HR (95% CI)** | **P value** | **HR (95% CI)** | **P value** |
| **cuCHG** | 588 (9.1) | 1.35 (1.27~1.43) | < 0.001 | 1.35 (1.27~1.44) | < 0.001 | 1.23 (1.15~1.31) | < 0.001 |
| Q1 | 104 (6) | Reference | — | Reference | — | Reference | — |
| Q2 | 119 (7.1) | 1.16 (0.89~1.5) | 0.283 | 1.14 (0.88~1.48) | 0.331 | 1.05 (0.8~1.36) | 0.738 |
| Q3 | 147 (9) | 1.38 (1.07~1.77) | 0.013 | 1.32 (1.03~1.7) | 0.031 | 1.1 (0.85~1.42) | 0.46 |
| Q4 | 218 (15.1) | 2.5 (1.98~3.16) | < 0.001 | 2.45 (1.94~3.11) | < 0.001 | 1.78 (1.39~2.28) | < 0.001 |
| P trend |  |  | < 0.001 |  | < 0.001 |  | < 0.001 |
| **CHG pattern groups** |  |  |  |  |  |  |  |
| Cluster 1 | 173 (6.3) | Reference | — | Reference | — | Reference | — |
| Cluster 2 | 288 (9.3) | 1.42 (1.18~1.71) | < 0.001 | 1.37 (1.13~1.65) | 0.001 | 1.13 (0.93~1.38) | 0.203 |
| Cluster 3 | 127 (19.4) | 2.99 (2.38~3.76) | < 0.001 | 2.95 (2.34~3.71) | < 0.001 | 2.06 (1.61~2.63) | < 0.001 |
| P trend |  | | < 0.001 |  | < 0.001 |  | < 0.001 |

Model 1: unadjusted; Model 2: adjusted for age, sex, education, residence, marriage, smoking status, drinking status; Model 3: fully adjusted for age, sex, education, residence, marriage, smoking status, drinking status, BMI, hypertension, hypertension treatment, dyslipidaemia, dyslipidaemia treatment, and nephropathy. Abbreviation: BMI, body mass index; CHG: Cholesterol, High-Density Lipoprotein, and Glucose; CI, confidence interval; CMM: Cardiometabolic Multimorbidity; HR, hazard ratio.

# **Table S5. Sensitivity analysis after excluding individuals with outliers**

| **Character** | **Event, n (%)** | **Model 1** | | **Model 2** | | **Model 3** | |
| --- | --- | --- | --- | --- | --- | --- | --- |
|  |  | **HR (95% CI)** | **P value** | **HR (95% CI)** | **P value** | **HR (95% CI)** | **P value** |
| **cuCHG** | 559 (8.9) | 1.46 (1.35~1.59) | < 0.001 | 1.47 (1.35~1.59) | < 0.001 | 1.31 (1.2~1.42) | < 0.001 |
| Q1 | 97 (5.8) | Reference | — | Reference | — | Reference | — |
| Q2 | 113 (6.8) | 1.16 (0.88~1.52) | 0.297 | 1.14 (0.87~1.49) | 0.357 | 1.05 (0.8~1.38) | 0.729 |
| Q3 | 148 (9.1) | 1.46 (1.13~1.89) | 0.004 | 1.4 (1.08~1.81) | 0.01 | 1.17 (0.9~1.53) | 0.228 |
| Q4 | 201 (14.9) | 2.56 (2.01~3.27) | < 0.001 | 2.51 (1.96~3.21) | < 0.001 | 1.84 (1.42~2.37) | < 0.001 |
| P trend |  |  | < 0.001 |  | < 0.001 |  | < 0.001 |
| **CHG pattern groups** |  |  |  |  |  |  |  |
| Cluster 1 | 161 (6.1) | Reference | — | Reference | — | Reference | — |
| Cluster 2 | 287 (9.3) | 1.5 (1.24~1.82) | < 0.001 | 1.45 (1.19~1.76) | < 0.001 | 1.21 (0.99~1.47) | 0.063 |
| Cluster 3 | 111 (19.3) | 3.12 (2.45~3.97) | < 0.001 | 3.08 (2.41~3.93) | < 0.001 | 2.15 (1.67~2.78) | < 0.001 |
| P trend |  | | < 0.001 |  | < 0.001 |  | < 0.001 |

Model 1: unadjusted; Model 2: adjusted for age, sex, education, residence, marriage, smoking status, drinking status; Model 3: fully adjusted for age, sex, education, residence, marriage, smoking status, drinking status, BMI, hypertension, hypertension treatment, dyslipidaemia, dyslipidaemia treatment, and nephropathy. Abbreviation: BMI, body mass index; CHG: Cholesterol, High-Density Lipoprotein, and Glucose; CI, confidence interval; CMM: Cardiometabolic Multimorbidity; HR, hazard ratio.

# **Table S6. Sensitivity analysis after additional covariate adjustment**

| **Character** | **Event, n (%)** | **Model 1** | | **Model 2** | | **Model 3** | |
| --- | --- | --- | --- | --- | --- | --- | --- |
|  |  | **HR (95% CI)** | **P value** | **HR (95% CI)** | **P value** | **HR (95% CI)** | **P value** |
| **cuCHG** | 593 (9.1) | 1.35 (1.27-1.43) | < 0.001 | 1.36 (1.27-1.44) | < 0.001 | 1.24 (1.16-1.33) | < 0.001 |
| Q1 | 104 (6) | Reference | — | Reference | — | Reference | — |
| Q2 | 119 (7.1) | 1.15 (0.88-1.50) | 0.298 | 1.13 (0.87-1.47) | 0.357 | 1.06 (0.82-1.39) | 0.653 |
| Q3 | 150 (9.1) | 1.40 (1.09-1.80) | 0.008 | 1.34 (1.04-1.72) | 0.022 | 1.15 (0.89-1.48) | 0.296 |
| Q4 | 220 (15.2) | 2.51 (1.98-3.16) | < 0.001 | 2.45 (1.93-3.10) | < 0.001 | 1.84 (1.44-2.37) | < 0.001 |
| P trend |  |  | < 0.001 |  | < 0.001 |  | < 0.001 |
| **CHG pattern groups** |  |  |  |  |  |  |  |
| Cluster 1 | 173 (6.3) | Reference | — | Reference | — | Reference | — |
| Cluster 2 | 292 (9.4) | 1.43 (1.19-1.73) | < 0.001 | 1.38 (1.14-1.67) | 0.001 | 1.17 (0.97-1.43) | 0.106 |
| Cluster 3 | 128 (19.4) | 2.99 (2.38-3.75) | < 0.001 | 2.94 (2.33-3.71) | < 0.001 | 2.1 (1.65-2.69) | < 0.001 |
| P trend |  | | < 0.001 |  | < 0.001 |  | < 0.001 |

Model 1: unadjusted; Model 2: adjusted for age, sex, education, residence, marriage, smoking status, drinking status; Model 3: fully adjusted for age, sex, education, residence, marriage, smoking status, drinking status, BMI, hypertension, hypertension treatment, dyslipidaemia, dyslipidaemia treatment, and nephropathy. Abbreviation: BMI, body mass index; CHG: Cholesterol, High-Density Lipoprotein, and Glucose; CI, confidence interval; CMM: Cardiometabolic Multimorbidity; HR, hazard ratio.
